# The emerging role of human TBK1 in virus-induced autophagy

**DOI:** 10.1080/15548627.2019.1580513

**Published:** 2019-02-22

**Authors:** Ahmad Liyana, Sancho-Shimizu Vanessa

**Affiliations:** a Department of Virology, Division of Medicine, Imperial College London, London, UK; b PAPRSB Institute of Health Sciences, Universiti Brunei Darussalam, Bandar Seri Begawan, Brunei Darussalam; c Department of Paediatrics, Division of Medicine, Imperial College London, London, UK

**Keywords:** Autophagy, CNS, HSV-1, infection, inflammation, interferon, TBK1

## Abstract

Recent studies have suggested a role for TBK1 in mediating inflammation through macroautophagy/autophagy. While its function in inducing interferon production in response to viral infection has been extensively studied, its role in antiviral autophagy is only beginning to be appreciated. Herein we discuss the role of this multifunctional protein in both antiviral IFN production and in cytoprotective autophagy during HSV-1 infection. Lastly, we highlight the potential implication of TBK1 in the management of inflammation through autophagy, particularly within the central nervous system.

TBK1 is a ubiquitous serine/threonine protein kinase traditionally known for its role in inducing type-I interferons (IFNs) downstream of TLR3-IFN and TMEM173/STING-IFN signalling pathways. A number of studies have also demonstrated the involvement of TBK1 in selective autophagy. Autophagy is a multistep process of degrading cellular contents, including toxic protein aggregates and intracellular pathogens. Autophagy can occur in a selective manner whereby an autophagy receptor, such as CALCOCO2/NDP52, OPTN (optineurin) or SQSTM1/p62, is recruited to capture target cargo. TBK1 has been recently highlighted as a key player in the activation of these selective receptors in the elimination of multiple human pathogens including herpes simplex virus-1 (HSV-1).

HSV-1 is the causative pathogen of herpes simplex encephalitis (HSE), which is characterized by necrotizing lesions in the central nervous system (CNS). *TBK1* mutations causing autosomal dominant *TBK1* deficiency have been identified in HSE patients resulting in impaired IFN production leading to enhanced cell death upon infection with HSV-1. As such, IFN plays a critical antiviral role during HSV-1 infection. Additionally, there is a growing appreciation for the antiviral effect of autophagy in the field. Our study reported 2 autophagy phenotypes that take place sequentially following HSV-1 infection in human dermal fibroblasts [1]. Early in infection, control fibroblasts form cytoplasmic LC3 puncta, followed by perinuclear LC3 formation later in infection. The early cytoplasmic autophagy response also occurs in viral-antigen-negative cells in the vicinity of infected cells. Interestingly, TBK1-deficient fibroblasts fail to trigger this early autophagy response upon HSV-1 infection. Furthermore, the early autophagy response appears to be an IFN-independent process, as TICAM1/TRIF-deficient fibroblasts, which have a complete impairment of HSV-1-induced IFN production, are able to induce it. We further demonstrated the protective effect of early autophagy induction such that induction of cytoplasmic LC3 puncta by rapamycin prior to HSV-1 infection leads to reduced cell mortality post-infection. However, this has no effect on viral replication, as viral titres with or without rapamycin remain the same. Conversely, the late perinuclear autophagy phenotype, which has been described by other groups and termed ‘nuclear envelope-derived autophagy’, appears to be a TBK1-independent event reflective of a general host stress response to viral infection.

The early autophagy response in HSV-1 infection has been documented previously, where its occurrence was linked to the presence of the HSV-1 double-stranded DNA genome in human foreskin fibroblasts and mouse macrophages. Furthermore, this autophagy induction was mediated by TMEM173/STING, an adaptor protein that functions downstream of multiple dsDNA sensors. It is generally thought that the HSV-1 genome is kept intact in its nucleocapsid through its journey towards the nucleus where viral replication will occur. While the mechanism by which the viral genome gains access to the cytoplasm and triggers autophagy induction is still unclear, a study has shown that it may be present in the cytoplasm as early as 2h following infection in human macrophages and a retinal epithelial cell line. Reportedly, the nucleocapsid can be degraded by proteasomes following cellular entry, which releases the HSV-1 dsDNA genome for TMEM173/STING- and TBK1-dependent IFN production. The dual role of the TBK1-dependent dsDNA signalling pathway in mediating both IFN and autophagy has been described in *Mycobacteria tuberculosis* infection, begging the question of whether a similar mechanism may be involved in the early autophagy response in HSV-1 infection. Further studies dissecting the molecular composition of the cytoplasmic LC3 puncta may help elucidate the key players that trigger this phenomenon.

Another interesting feature of the early autophagy response was that it occurs in cells neighboring infected cells, suggesting a possible paracrine-mediated induction of autophagy during HSV-1 infection. Paracrine-mediated autophagy has been documented in human placenta where it promotes cytoprotection against multiple viral infections. Maternal trophoblasts are able to confer resistance to viruses in various non-trophoblastic cell types by upregulating autophagy in these cells. The significance of paracrine-mediated autophagy in HSV-1 infection and how it is induced is still unclear. Exploring the triggers of autophagy upon infection and the precise mechanism of induction in these neighboring cells will help illuminate the relevance of this mode of autophagy.

Finally, elegant mouse models of HSV-1 infection have demonstrated that autophagy is the preferred antiviral response against HSV-1 in the CNS over IFN, as it encourages the survival of non-renewable neuronal cells. Interestingly, numerous human *TBK1* mutations have been identified in patients with neurodegenerative diseases such as amyotrophic lateral sclerosis (ALS), as well as frontotemporal dementia (FTD), highlighting a role for TBK1 in the CNS. This finding appears to associate TBK1 with neuroinflammation via dysregulation of autophagy. Given the implication of human TBK1 in anti-inflammatory and cytoprotective autophagy, it is tempting to speculate on the relevance of TBK1 in controlling the neurological damage caused by HSV-1-induced inflammation and its role in the long-term outcomes of HSE. While animal models of ALS benefit from rapamycin treatment, future exploration of TBK1 function in neuroprotective autophagy may provide more targeted therapeutic options for these diseases via selective autophagy modulation.

## References

[1] Ahmad L, Mashbat B, Leung C, et al Human TANK-binding kinase 1 is required for early autophagy induction upon herpes simplex virus 1 infection. J Allergy Clin Immunol. 2019 Feb;143(2):765–769.e7. doi:10.1016/j.jaci.2018.09.013. Epub 2018 Oct 5–. 30296527

